# Therapeutics of Infancy and Childhood

**Published:** 1904-11

**Authors:** 


THERAPEUTICS OF INFANCY AND CHILDHOOD. By A. Jacobi, M. D., LL. D. Third edition. J. B. Lippincott Company, Publishers, Philadelphia and London. Price, cloth, $3.50. 1903.

This work has reached the third edition within eight years, attesting its popularity with the profession. The general plan of the book is most excellent, being divided into fifteen sections or articles, as follows: I. Feeding of Sick Children. II. General Therapeutics. III. Treatment of the Newly-born. IV. Disease of the Blood and Constitution. V. Infectious Diseases. VI. Disease of the Nervous System. VII. Diseases of the Digestive Organs. VIII. Diseases of the Genito-Urinary Organs. IX. Diseases of the Respiratory Organs. X. Diseases of the Organs of Circulation. XI. Diseases of the Skin. XII. Diseases of Muscles. XIII. Diseases of the Bones and Joints. XIV. Diseases of the Ear. XV. Diseases of the Eye. The author devotes more space to the discussion of infectious diseases than to any other division of the book. In this division he treats of tuberculosis, syphilis, intermittent fever, typhoid fever, typhus, relapsing fever, Weil's disease, epidemic cerebro-spinal meningitis, glandular fever, catarrhal fever, Asiatic cholera, dysentery, scarlatina, measles, röthelm, mumps, variola, varicella, vaccination, erysipelas, diphtheria, rheumatism, influenza, pertussis. The placing of some of these subjects in the list of infectious diseases will excite some surprise, but as our knowledge of pathology and etiology advances it is found that many diseases are being added to the list of those caused by germs. The entire volume is excellent in every way, but article II, subject, treatment of the newly-born, is especially interesting and instructive, dealing, as it does, with subjects coming frequently under the observation of every general practitioner; many of these subjects not being too well understood by the average practitioner. S. E. H.

